# Reduced rapid eye movement sleep in late middle-aged and older apolipoprotein E **ɛ**4 allele carriers

**DOI:** 10.1093/sleep/zsae094

**Published:** 2024-04-18

**Authors:** Claire André, Marie-Ève Martineau-Dussault, Andrée-Ann Baril, Nicola Andrea Marchi, Véronique Daneault, Dominique Lorrain, Carol Hudon, Célyne H Bastien, Dominique Petit, Cynthia Thompson, Judes Poirier, Jacques Montplaisir, Nadia Gosselin, Julie Carrier

**Affiliations:** Center for Advanced Research in Sleep Medicine, Hôpital du Sacré-Coeur de Montréal, Recherche CIUSSS NIM, Montreal, QC, Canada; Department of Psychology, Université de Montréal, Montreal, QC, Canada; Center for Advanced Research in Sleep Medicine, Hôpital du Sacré-Coeur de Montréal, Recherche CIUSSS NIM, Montreal, QC, Canada; Department of Psychology, Université de Montréal, Montreal, QC, Canada; Center for Advanced Research in Sleep Medicine, Hôpital du Sacré-Coeur de Montréal, Recherche CIUSSS NIM, Montreal, QC, Canada; Department of Medicine, Université de Montréal, Montreal, QC, Canada; Center for Advanced Research in Sleep Medicine, Hôpital du Sacré-Coeur de Montréal, Recherche CIUSSS NIM, Montreal, QC, Canada; Department of Psychology, Université de Montréal, Montreal, QC, Canada; Center for Investigation and Research in Sleep, Department of Medicine, Lausanne University Hospital and University of Lausanne, Lausanne, Switzerland; Laboratory for Research in Neuroimaging, Department of Clinical Neurosciences, Lausanne University Hospital and University of Lausanne, Lausanne, Switzerland; Center for Advanced Research in Sleep Medicine, Hôpital du Sacré-Coeur de Montréal, Recherche CIUSSS NIM, Montreal, QC, Canada; Department of Psychology, Université de Montréal, Montreal, QC, Canada; Research Centre on Aging, University Institute of Geriatrics of Sherbrooke, CIUSSS de l’Estrie-CHUS, Sherbrooke, QC, Canada; Department of Psychology, Université de Sherbrooke, Sherbrooke, QC, Canada; CERVO Brain Research Centre, Institut Universitaire en Santé Mentale de Québec, Québec City, QC, Canada; School of Psychology, Université Laval, Québec City, QC, Canada; CERVO Brain Research Centre, Institut Universitaire en Santé Mentale de Québec, Québec City, QC, Canada; School of Psychology, Université Laval, Québec City, QC, Canada; Center for Advanced Research in Sleep Medicine, Hôpital du Sacré-Coeur de Montréal, Recherche CIUSSS NIM, Montreal, QC, Canada; Department of Psychiatry, Université de Montréal, Montréal, QC, Canada; Center for Advanced Research in Sleep Medicine, Hôpital du Sacré-Coeur de Montréal, Recherche CIUSSS NIM, Montreal, QC, Canada; Department of Psychiatry, McGill University, Montreal, QC, Canada; Douglas Mental Health University Institute, CIUSSS de l’Ouest-de-l’Ile-de-Montréal, Verdun, QC, Canada; Center for Advanced Research in Sleep Medicine, Hôpital du Sacré-Coeur de Montréal, Recherche CIUSSS NIM, Montreal, QC, Canada; Department of Psychiatry, Université de Montréal, Montréal, QC, Canada; Center for Advanced Research in Sleep Medicine, Hôpital du Sacré-Coeur de Montréal, Recherche CIUSSS NIM, Montreal, QC, Canada; Department of Psychology, Université de Montréal, Montreal, QC, Canada; Center for Advanced Research in Sleep Medicine, Hôpital du Sacré-Coeur de Montréal, Recherche CIUSSS NIM, Montreal, QC, Canada; Department of Psychology, Université de Montréal, Montreal, QC, Canada

**Keywords:** *APOE4*, REM sleep, sleep, aging, mild cognitive impairment, polysomnography, Alzheimer’s disease, dementia

## Abstract

**Study Objectives:**

Apolipoprotein E ɛ4 (*APOE4*) is the strongest genetic risk factor for Alzheimer’s disease (AD). In addition, *APOE4* carriers may exhibit sleep disturbances, but conflicting results have been reported, such that there is no clear consensus regarding which aspects of sleep are impacted. Our objective was to compare objective sleep architecture between *APOE4* carriers and non-carriers, and to investigate the modulating impact of age, sex, cognitive status, and obstructive sleep apnea (OSA).

**Methods:**

A total of 198 dementia-free participants aged >55 years old (mean age: 68.7 ± 8.08 years old, 40.91% women, 41 *APOE4* carriers) were recruited in this cross-sectional study. They underwent polysomnography, *APOE4* genotyping, and a neuropsychological evaluation. ANCOVAs assessed the effect of *APOE4* status on sleep architecture, controlling for age, sex, cognitive status, and the apnea–hypopnea index. Interaction terms were added between *APOE4* status and covariates.

**Results:**

Rapid eye movement (REM) sleep percentage (*F* = 9.95, *p* = .002, η_p_^2^ = 0.049) and duration (*F* = 9.23, *p* = .003, η_p_^2^ = 0.047) were lower in *APOE4* carriers. The results were replicated in a subsample of 112 participants without moderate-to-severe OSA. There were no significant interactions between *APOE4* status and age, sex, cognitive status, and OSA in the whole sample.

**Conclusions:**

Our results show that *APOE4* carriers exhibit lower REM sleep duration, including in cognitively unimpaired individuals, possibly resulting from early neurodegenerative processes in regions involved in REM sleep generation and maintenance.

## Graphical Abstract

**Graphical Abstract:**
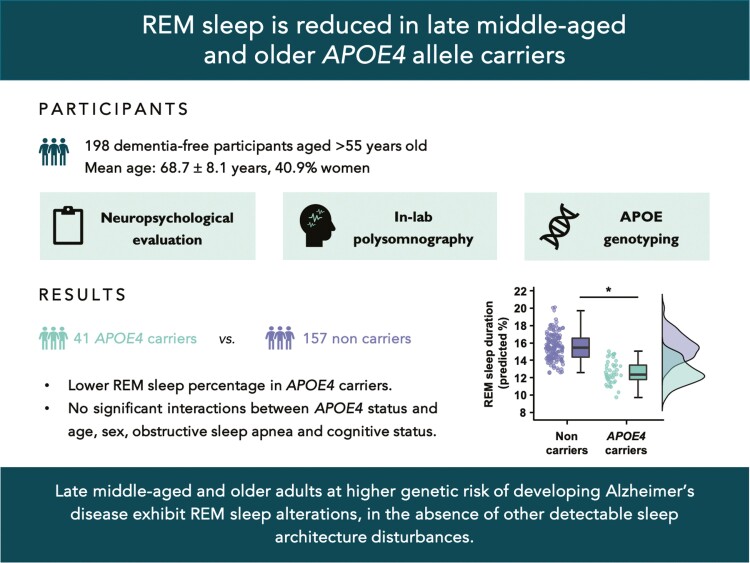

**Statement of Significance**

Apolipoprotein E ɛ4 (*APOE4*) carriers, who are at greater risk of developing Alzheimer’s disease, may exhibit sleep disturbances. However, there is no consensus on which aspects of sleep are disrupted, notably in *APOE4* carriers without cognitive deficits. In 198 dementia-free late middle-aged and older participants, we show that *APOE4* carriers exhibited lower rapid eye movement (REM) sleep proportion and duration than non-carriers. In the whole sample, there was no significant interaction with age, sex, cognitive status, or sleep apnea. This suggests that sleep architecture alterations are detectable in *APOE4* carriers, even in the absence of cognitive deficits. Future studies should investigate the neural mechanisms underlying REM-sleep disturbances in *APOE4* carriers and assess whether they worsen in those developing cognitive deficits.

Approximately 15%–20% of the general population present with at least one copy of the ε4 allele of the *Apolipoprotein E* (*APOE4*) gene [1]. *APOE4* is the strongest genetic risk factor for sporadic Alzheimer’s disease (AD) [1, 2]. Compared to non-carriers, *APOE4* carriers present with earlier cognitive decline as well as deficits of greater magnitude in various cognitive domains, including memory [3, 4]. While the mechanisms by which *APOE4* confers a greater risk of developing AD are not fully understood, they seem intimately associated with AD pathological processes. Indeed, *APOE4* is related to increased amyloid production and lower clearance, as well as greater tau-related neurodegeneration and neuroinflammation [5–9]. Interestingly, the links between *APOE4* and AD risk may be stronger in women compared to men, possibly because of greater tau pathology levels [10, 11].

Sleep disturbances are frequent in older individuals, and poor sleep has been associated with cognitive decline and a higher risk of developing dementia. Indeed, in older people without cognitive deficits, sleep becomes shorter, lighter, more fragmented, and the prevalence of sleep disorders such as obstructive sleep apnea (OSA) increases [12, 13]. In patients with AD dementia or mild cognitive impairment (MCI), these changes are even more significant [14, 15]. Better understanding the aspects of sleep that are disrupted before the onset of dementia, in individuals at higher risk of developing AD, is crucial to clarify the early manifestations associated with the development of neurodegenerative processes.

An emerging literature suggests that *APOE4* carriers may exhibit more severe sleep disturbances than non-carriers. Several studies suggest that *APOE4* carriers exhibit shorter, more fragmented, and less efficient sleep [16–20], as well as lower rapid eye movement (REM) sleep duration [17] and a greater risk of OSA [21, 22]. However, conflicting results have also been reported. For example, a recent meta-analysis regrouping 6508 participants (1901 OSA cases and 4607 controls) has reported that *APOE4* is not significantly associated with OSA [23]. In addition, other studies have not observed significant differences in subjective sleep variables [20] or actigraphic measures of sleep consolidation/fragmentation [24] between *APOE4* carriers and non-carriers. Overall, there is no clear consensus regarding which aspects of sleep are impacted in older *APOE4* carriers, notably in older adults without cognitive deficits. Methodological differences are likely to explain part of this heterogeneity, as various methods have been used to assess sleep (i.e. questionnaires, actigraphy, and polysomnography), in different study populations (i.e. patients with dementia, MCI, and cognitively normal older adults) and sometimes very small samples (i.e. ≤10 *APOE4* carriers in previous studies using polysomnography, except one study including 555 male *APOE4* carriers, but no women [25]). Another hypothesis is that sleep differences between *APOE4* carriers and non-carriers may be influenced by demographical characteristics and comorbidities. Notably, previous studies have not systematically assessed and controlled for OSA, which is very prevalent in older populations, yet largely underdiagnosed [13].

Our objective was to identify the alterations of sleep architecture in *APOE4* carriers, and the impact of potential modulating factors (i.e. age, sex, cognitive status, and OSA severity) on these differences, in order to identify subgroups whose sleep quality may potentially be more vulnerable to *APOE4* carriage. We expected *APOE4* carriers to exhibit altered sleep architecture (e.g. lower REM-sleep duration and greater sleep fragmentation) compared to non-carriers, especially in subgroups more vulnerable to cognitive decline (e.g. older participants, women, and participants with OSA).

## Materials and Methods

### Participants

We recruited 198 participants between 2012 and 2019 from local memory and sleep clinics and the community in Montreal and Sherbrooke (Canada). Participants were included in the context of four protocols between 2012 and 2020, all approved by institutional ethics committees (n° 2012-697, 12-13-008, 2010-468, and MP-32-2018-1537). A written informed consent was obtained from each participant prior to the examinations, according to the declaration of Helsinki. Between 2012 and 2016, 149 participants were recruited in Montreal as part of three protocols on aging and MCI. Between 2018 and 2020, 49 participants were recruited from two sites as part of a multicentric project (*n* = 15 in Montreal and *n* = 34 in Sherbrooke). The recruitment process consisted of a phone screening followed by an in-person interview, a neuropsychological evaluation, and an in-lab polysomnographic recording. All participants were aged over 55 years old, fluent in French or English, had a minimum of 7 years of education, and preserved autonomy in daily life. Exclusion criteria encompassed the presence or history of major neurological disorders (e.g. dementia, epilepsy, traumatic head injury, or encephalopathy), psychiatric disorders (e.g. diagnosed major depression or anxiety), sleep disorders other than OSA (e.g. insomnia, periodic limb movement disorder, REM-sleep behavior disorder), cerebrovascular or pulmonary diseases (e.g. history of stroke and chronic obstructive pulmonary disease), uncontrolled diabetes or hypertension, body mass index greater than 40 km/m^2^, drug or alcohol abuse, heavy consumption of caffeinated beverages, and the use of medications affecting sleep, cognition, or brain functioning (e.g. antidepressants, hypnotics, and opioids).

### Neuropsychological evaluation and questionnaires

Participants underwent a comprehensive neuropsychological evaluation of global cognitive functioning, attention and processing speed, executive functioning, learning and memory, language, and visuospatial abilities [26]. Cognitive diagnosis was established by consensus between senior neuropsychologists and neurologists, based on cognitive performance. Participants were classified as (1) cognitively unimpaired (*n* = 109) if cognitive performance was preserved in all domains, or (2) having amnestic MCI (aMCI; *n* = 89) if at least the memory domain was altered (i.e. defined as presenting at least two *Z*-scores ≤1.5 SD in the memory domain, or an MoCA score < 26 accompanied by two *Z*-scores ≤1.5 SD in several distinct cognitive domains, including at least one test in the memory domain) [26]. Participants with non-amnestic MCI were not included in the present study (*n* = 28).

### Polysomnographic recording

Participants underwent an in-lab polysomnography, encompassing at least 12 electrodes placed on the scalp according to the international 10–20 system referenced to the mastoids (F3, F4, C3, C4, T3, T4, T5, T6, P3, P4, O1, and O2), as well as electrooculogram, electrocardiogram, chin, and leg electromyogram electrodes, oronasal canula and thermistors, thoraco-abdominal belts, and a finger pulse oximeter. Two different polysomnography systems were used: a Grass system between 2012 and 2016 (*n *= 155 participants; bandpass 0.3–100 Hz; signal digitized at a sampling rate of 256 Hz using Harmonie software; Stellate Systems, Montreal, Quebec, Canada), and a Natus system after 2019 (*n* = 43 participants; bandpass 0.3–200 Hz, digitized at a sampling rate of 512 Hz) (Brain Monitor, Trex, and Embla NDx). Recordings were scored by certified sleep technologists in 30-second epochs according to international criteria [27]. We calculated standard sleep variables (i.e. sleep duration, latency, efficiency, and the duration of wake after sleep onset), the duration and proportion of each sleep stage and sleep apneas and hypopneas. Sleep apneas were defined as drops of ≥90% of airflow for a minimum of 10 seconds, and hypopneas were defined as a ≥30% reduction of airflow for a minimum of 10 seconds, followed by either a cortical arousal or a ≥3% oxygen desaturation [27]. We computed the apnea–hypopnea index (AHI) as the number of apneas and hypopneas per hour of sleep.

### *APOE* genotyping

*APOE* genotyping was performed using standard protocols, as previously described [28]. Briefly, DNA was extracted from buffy coats obtained from blood samples using the Qiagen EZ1 DNA Kit. Pyrosequencing was conducted to identify both *APOE* single-nucleotide polymorphism sites, allowing us to determine ε2, ε3, and ε4 isoforms [28]. In our sample, 157 participants were non-*APOE4* carriers (1 participant was ε2:2, 24 were ε2:3, 132 were ε3:3) and 41 were *APOE4* carriers (1 was ε2:4, 35 were ε3:4, and 5 were ε4:4).

### Statistical analyses

We compared demographic, cognitive, and sleep characteristics between *APOE4* carriers and non-carriers using Student’s *t* tests for continuous variables and chi-squared tests for categorical variables. Then, between-group differences in sleep architecture (i.e. total sleep time, sleep onset latency, sleep efficiency, wake after sleep onset, sleep stages percentages, and REM-sleep latency) were assessed using type II ANCOVAs, with *APOE4* status added as a factor. Age, sex, cognitive status (i.e. cognitively unimpaired or aMCI), and the AHI were added as covariates. For significant ANCOVA models, we then tested interactions between *APOE4* status and covariates (i.e. age, sex, cognitive status, and the AHI) on sleep architecture variables. For the sake of completeness, we also replicated these analyses in subgroups stratified by age, sex, cognitive status, and OSA diagnosis. These results are available in Supplementary Material.

Secondary sensitivity analyses were performed to ensure the robustness of our results. First, we verified whether significant results obtained with the percentage of sleep stages were similar when considering sleep stages duration expressed in minutes, or when controlling for the AHI computed specifically during REM sleep rather than total sleep time. Second, the analyses were replicated in a subsample of 112 participants without moderate-to-severe OSA (i.e. removing those with an AHI > 15), including 24 *APOE4* carriers and 88 non-carriers. Third, we checked whether significant *APOE*-related sleep characteristics were associated with other sleep variables, by performing multiple regression analyses between sleep architecture variables in the whole sample, as well as in *APOE4* carriers only, controlling for age, sex, cognitive status, and the AHI.

All non-normal variables (i.e. sleep onset latency, REM-sleep latency, N1 and N3 percentages, wake after sleep onset, the number of awakenings, and the AHI) were log-transformed prior to statistical analyses. Significance was set to *p* < .05, and we have applied a Bonferroni correction to account for the number of statistical analyses performed.

## Results

### Participants characteristics

The sample was composed of 198 participants (mean age: 68.7 ± 8.08 years old, 40.91% women), including 41 *APOE4* carriers (mean age: 69.9 ± 7.16 years old, 48.78% women) and 157 non-carriers (mean age: 68.38 ± 8.3 years old, 38.85% women). Their demographic and clinical characteristics are summarized in Table 1. *APOE4* carriers and non-carriers did not differ in terms of demographics, including age, sex ratio, education, the proportion of individuals with MCI in each group, body mass index, and AHI. Moreover, the two groups did not differ in terms of sleep architecture, except for REM-sleep duration (expressed in minutes or as a percentage of total sleep time), which was significantly lower in *APOE4* carriers as compared to non-carriers (REM-sleep min: *T* = 2.97, *p* = .003, Cohen’s *d* = 0.52; REM-sleep percentage: *T* = 2.88, *p* = .004, Cohen’s *d* = 0.51; Table 1).

**Table 1.** Participants Characteristics

Variable	Whole sample (*n* = 198)		Non-carriers (*n* = 157)		*APOE4* carriers (*n* = 41)		Between-group differences		
	Mean	*SD*	Mean	*SD*	Mean	*SD*	*T* or χ^2^	*P*	Cohen’s *d*
*Demographics and cognition*									
Age: years	68.7	8.08	68.38	8.3	69.9	7.16	−1.07	.29	−0.19
Sex: nb (%) of women	81 (40.91)		61 (38.85)		20 (48.78)		1.33	.25	-
Education: years	14.75	3.74	14.68	3.74	15.02	3.77	−0.52	.60	−0.09
Body mass index: kg/m^2^	26.92	3.87	27.02	4.01	26.53	3.28	0.72	.47	0.13
Cognitive status: nb (%) of individuals with aMCI	89 (44.95)		66 (42.04)		23 (56.10)		2.60	.11	-
MoCA score ^1^	26.5	2.73	26.55	2.72	26.31	2.83	0.48	.63	0.09
*Sleep*									
Total sleep time: min	344.65	69.52	349.32	66.76	326.77	77.53	1.86	.06	0.33
Sleep latency: min	15.34	18.9	15.03	19.12	16.54	18.21	−0.76	.45	−0.13
Sleep efficiency: %	76.6	12.35	77.28	11.48	73.98	15.09	1.53	.13	0.27
Nighttime awakenings: nb	41.41	23.74	42.36	23.21	37.78	25.64	1.88	.06	0.33
WASO: min	102.93	53.16	100.61	50.95	111.83	60.77	−0.82	.41	−0.15
N1-sleep: min	76.7	44.13	78.79	44.76	68.67	41.15	1.31	.19	0.23
N1-sleep: % TST	22.52	12.37	22.87	12.47	21.19	12.04	0.8	.42	0.14
N2-sleep: min	186.98	56.65	187.39	55.26	185.39	62.4	0.2	.84	0.04
N2-sleep: % TST	53.83	10.37	53.25	10.25	56.05	10.64	−1.55	.12	−0.27
N3-sleep: min	28.21	29.66	27.87	30.07	29.5	28.35	−0.31	.76	−0.06
N3-sleep: % TST	8.72	9.6	8.35	9.38	10.16	10.42	−0.79	.43	−0.14
REM-sleep: min	52.77	23.64	55.26	24.01	43.21	19.61	2.97	**.003**	0.52
REM-sleep: % TST	14.93	5.92	15.54	5.95	12.6	5.25	2.88	**.004**	0.51
REM-sleep latency: min ^2^	124.26	78.3	118.21	71.98	148.64	97.11	−1.65	.10	−0.29
Total AHI: nb/h	18.35	17.12	19.03	17.18	15.78	16.89	0.85	.40	0.15
REM-sleep AHI: nb/h	22.45	20.63	22.10	20.66	22.82	20.78	−0.11	.91	−0.02
Moderate-to-severe OSA: nb (%)	86 (43.43)		69 (43.95)		17 (41.46)		0.08	.78	-

Differences between *APOE4* carriers and non-carriers were assessed using Student *t*-tests for continuous variables and chi-squared tests for categorical variables. Results in bold indicate significant differences at the *P*<.05 uncorrected level.

^1^Missing data for 17 participants (12 non-carriers and 5 *APOE4* carriers).

^2^No data for 2 *APOE4* carriers due to lack of REM sleep.

Abbreviations: APOE4, ε4 allele of the Apolipoprotein E; AHI, apnea–hypopnea index; h, hour; nb, number; NREM, non-rapid eye movement; OSA, obstructive sleep apnea; REM, rapid eye movement; SD, standard deviation, TST, total sleep time, WASO, wake after sleep onset.

### Differences in sleep architecture according to *APOE4* status

**Figure 1.**
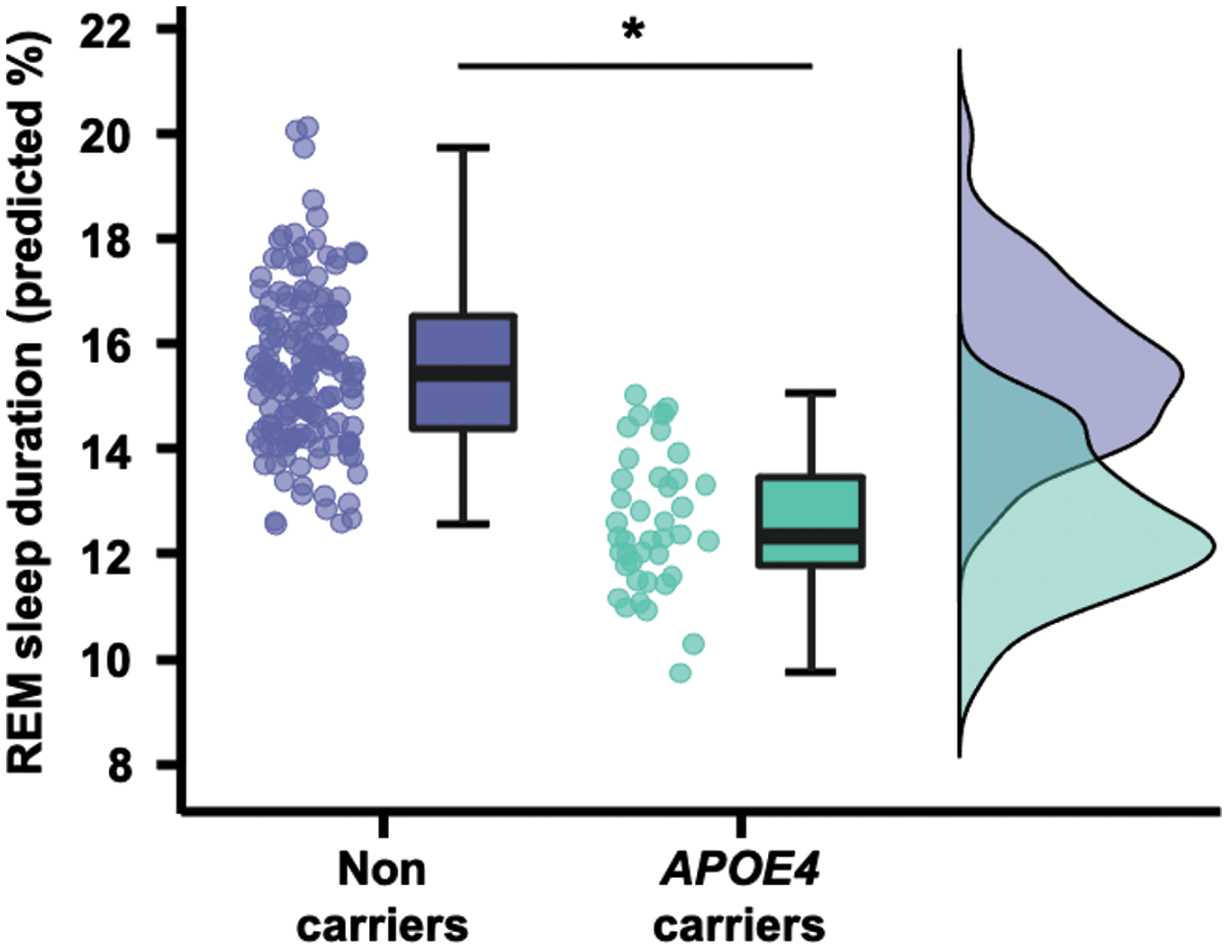
Differences in REM sleep proportion according to *APOE4* status. Raincloud plot showing predicted REM sleep proportion values (taking into account age, sex, cognitive status and the apnea-hypopnea index, expressed as a percentage of total sleep time) according to *APOE4* status. Boxplots represent medians, interquartile range and associated confidence intervals. Abbreviations: APOE4, ε4 allele of the Apolipoprotein E; REM, rapid eye movement.

We then performed ANCOVAs to assess the effect of *APOE4* status on sleep architecture variables, controlling for age, sex, the AHI, and cognitive status (i.e. cognitively unimpaired vs. aMCI). Only REM-sleep percentage significantly differed between *APOE4* carriers and non-carriers, such that *APOE4* carriers presented with lower REM-sleep proportion (*F* = 9.90, *p* = .002, η_p_^2^ = 0.049; Table 2 and Figure 1).

**Table 2.** Impact of *APOE*4 Status on Sleep Architecture in the Whole Sample

Dependent variable	Sum of squares	Degrees of freedom	Mean difference (95% CI)	*F*	*P*	η_p_^2^
Total sleep time (min)	12 494.63	1	19.86 (−4.41 to 44.12)	2.61	.11	0.01
Log(Sleep latency) (min)	0.04	1	−0.036 (−0.19 to 0.11)	0.23	.63	0.001
Sleep efficiency (%)	335.54	1	3.25 (−1.07 to 7.57)	2.21	.14	0.01
Log(Nb of awakenings) (nb)	0.05	1	0.04 (−0.03 to 0.11)	1.27	.26	0.007
Log(WASO) (min)	0.06	1	−0.04 (−0.13 to 0.04)	0.95	.33	0.005
Log(N1 sleep proportion) (% TST)	7.02e^−5^	1	−0.001 (−0.07 to 0.07)	0.002	.97	9.47e^−6^
N2 sleep proportion (% TST)	165.45	1	−2.29 (−5.73 to 1.16)	1.71	.19	0.01
Log(N3 sleep proportion) (% TST)	0.02	1	−0.02 (−0.17 to 0.12)	0.10	.75	5.25e^−4^
**REM sleep proportion (% TST)**	**320.36**	**1**	**3.18 (1.19 to 5.17)**	**9.90**	**.002**	**0.049**
Log(REM-sleep latency) (min)	0.14	1	−0.07 (−0.16 to 0.02)	2.14	.15	0.01

Results of type II ANCOVAs performed with each sleep architecture variable as dependent variables, separately, *APOE4* status as a factor, and age, sex, cognitive status, and log-transformed apnea–hypopnea index as covariates. Results in bold survived a Bonferroni correction for multiple comparisons (*p* = .05/number of comparisons = .05/10 = .005).

Abbreviations: APOE4, ε4 allele of the Apolipoprotein E; min, minutes; η_p_^2^, partial eta squared; NREM, non-rapid eye movement; REM, rapid eye movement; TST, total sleep time; WASO, wake after sleep onset.

To ensure the robustness of the association between *APOE4* status and REM sleep percentage, we performed sensitivity analyses. First, we replicated the main ANCOVA assessing the effect of *APOE4* on REM sleep, using REM sleep duration expressed in minutes as the dependent variable, rather than expressed as a percentage of total sleep time, controlling for the same covariates. We also observed a significant reduction of REM-sleep duration in *APOE4* carriers (*F* = 9.23, *p* = .003, η_p_^2^ = 0.047; Supplementary Table S1).

Second, adding the AHI computed specifically during REM sleep (instead of total sleep time) as a covariate confirmed the associations between *APOE4* status and REM sleep percentage (*F* = 8.93, *p* = .003, η_p_^2^ = 0.045) and duration (*F* = 9.23, *p* = .003, η_p_^2^ = 0.047; Supplementary Table S2).

Third, as OSA is known to affect sleep architecture, we replicated the main analyses in a subsample of 112 participants without moderate-to-severe OSA. As in the whole sample, REM-sleep duration was lower in *APOE4* carriers without moderate-to-severe OSA (*F* = 8.14, *p* = .005, η_p_^2^ = 0.071; Table 3).

**Table 3.** ANCOVAs Assessing the Impact of *APOE*4 Status on Sleep Architecture in the Subsample of 112 Participants Without Moderate-to-Severe OSA

Dependent variable	Sum of squares	Degrees of freedom	Mean difference (95%CI)	*F*	*P*	η_p_^2^
Total sleep time (min)	4893.86	1	16.33 (−15.54 to 48.20)	1.03	.31	0.01
Log(sleep latency) (min)	9.29e^−4^	1	−0.036 (−0.19 to 0.11)	0.005	.94	<0.001
Sleep efficiency (%)	156.01	1	2.02 (−2.58 to 8.41)	1.11	.30	0.01
Log(Nb of awakenings) (nb)	0.006	1	0.02 (−0.065 to 0.10)	0.19	.67	0.002
Log(WASO) (min)	0.02	1	−0.04 (−0.16 to 0.085)	0.34	.56	0.003
Log(N1 sleep proportion) (% TST)	0.005	1	−0.02 (−0.11 to 0.08)	0.11	.75	0.001
N2 sleep proportion (% TST)	204.18	1	−3.34 (−7.49 to 0.82)	2.54	.11	0.02
Log(N3 sleep proportion) (% TST)	0.04	1	0.05 (−0.16 to 0.25)	0.22	.64	0.002
**REM-sleep proportion (% TST)**	**241.62**	**1**	**3.63** **(1.11 to 6.15)**	**8.14**	**.005**	**0.071**
Log(REM-sleep latency) (min)	0.09	1	−0.07 (−0.18 to 0.04)	1.63	.20	0.02

Results of type II ANCOVAs performed with each sleep architecture variables as dependent variables, separately, and *APOE4* status as a factor, controlling for age, sex, cognitive status, and log-transformed AHI. Results in bold survive a Bonferroni correction for multiple comparisons (*p* = .05/number of comparisons = .05/10 = .005).

Abbreviations: APOE4, ε4 allele of the Apolipoprotein E; min, minutes; η_p_^2^, partial eta squared; NREM, non-rapid eye movement; REM, rapid eye movement; TST, total sleep time; WASO, wake after sleep onset.

Fourth, we verified whether REM sleep percentage was significantly associated with other sleep architecture variables. In the whole sample, we found that lower REM sleep percentage was robustly associated with lower total sleep time (*β* = 0.33, *p* < .001), lower sleep efficiency (*β* = 0.44, *p* < .001), greater wake after sleep onset (*β* = −0.35, *p* < .001), and greater percentage of N1 sleep (*β* = −0.25, *p* = .005; Supplementary Table S3). In the group of *APOE4* carriers only, the associations between lower REM sleep and both lower total sleep time (*β* = 0.52, *p* < .001) and lower sleep efficiency (*β* = 0.55, *p* < .001) remained significant, while the link with greater wake after sleep onset duration became a trend (*β* = −0.31, *p* = .07; Supplementary Table S3).

### Impact of demographics and comorbidities on REM-sleep percentage

We added interaction terms in the ANCOVA model to test whether *APOE4* status interacted with potential modulating factors (i.e. cognitive status, age, sex, and the AHI) on REM-sleep percentage and duration in the whole sample. Cognitive status, sex, age, and the AHI did not significantly interact with *APOE4* status in predicting REM-sleep percentage (Table 4) or duration (Supplementary Table S1).

**Table 4.** Interactions Between *APOE4* Status and Modulating Factors on the Prediction of REM-Sleep Percentage

Cases	Sum of squares	Degrees of freedom	*F*	*P*	η_p_^2^
APOE4 status	320.36	1	9.84	.002	0.05
Age	54.67	1	1.68	.2	0.01
Sex	2.59	1	0.08	.78	0.0004
Cognitive status	3.25	1	0.1	.75	0.001
log(AHI)	374.45	1	11.5	.001	0.06
APOE4*age	6.39	1	0.2	.66	0.001
APOE4*sex	3.77	1	0.12	.73	0.001
APOE4*cognitive status	73.31	1	2.25	.14	0.01
APOE4*log(AHI)	20.38	1	0.63	.43	0.003
Residuals	*6121.17*	*188*			

Type II ANCOVA testing the interactions between *APOE4* status and covariates (i.e. age, sex, cognitive status, and the AHI) on REM-sleep percentage in the whole sample. No significant interaction was observed.

Abbreviations: AHI, apnea–hypopnea index; APOE4, ε4 allele of the Apolipoprotein E; η_p_^2^, partial eta squared; REM, rapid eye movement.

For the sake of completeness, we replicated the analyses in subgroups stratified by age, sex, cognitive status, and OSA diagnosis (Supplementary Figure S1 and Table S4). We found that the effect of *APOE4* on REM-sleep percentage was (1) significant in the older group (*F* = 8.47, *p* = .005, η_p_^2^ = 0.08), and a trend in the younger group (*F* = 3.45, *p* = .066, η_p_^2^ = 0.036), (2) significant in women (*F* = 7.1, *p* = .01, η_p_^2^ = 0.085), and a trend in men (*F* = 11.7, *p* = .056, η_p_^2^ = 0.032), (3) significant in cognitively unimpaired (*F* = 11.7, *p* < .001, η_p_^2^ = 0.10) but not in aMCI participants, and (4) significant in both participants with (*F* = 4.1, *p* = .046, η_p_^2^ = 0.049) and without (*F* = 8.1, *p* = .005, η_p_^2^ = 0.07) OSA.

## Discussion

The present study aimed at comparing sleep architecture in late middle-aged and older participants carrying the ε4 allele of the *APOE* gene, who are at greater risk of developing sporadic AD, compared to non-carriers. Our results show that REM sleep duration and percentage were significantly lower in *APOE4* carriers, and that age, sex, cognitive status, and the presence of moderate-to-severe OSA did not significantly interact with *APOE4* status in predicting REM sleep. This suggests that older adults at higher genetic risk of developing AD are prone to exhibit REM-sleep alterations, in the absence of other detectable differences in sleep architecture, compared to controls.

If increasing age in the middle years of life has been associated with a reduction of REM sleep duration [29], REM sleep alterations are more marked in pathological aging and worsen with disease severity. Indeed, patients with dementia exhibit a reduction of REM sleep duration, as well as a global slowing of REM sleep EEG rhythms [15]. Interestingly, the reduction of REM sleep duration is already detectable in predementia stages. Patients with MCI exhibit shorter REM sleep compared to controls, especially those who will convert to AD [17, 30, 31]. The results of these clinical studies are consistent with animal studies showing that REM-sleep duration is lower in mice models of amyloid and tau pathologies compared to control mice [32, 33]. Interestingly, a previous study has shown a greater reduction of REM sleep in MCI patients who were *APOE4* carriers, compared to non-carriers [17]. Our results complement this observation in a larger sample of late middle-aged and older adults, notably in cognitively unimpaired individuals.

Furthermore, we found that *APOE4* genotype had no effect on other sleep stages. This result is in line with previous studies investigating the effect of *APOE4* on sleep architecture, which did not report significant effects on N3 sleep [17, 20]. One previous study has reported higher N3 sleep in 40 older men carrying two copies of the ɛ4 alleles, compared to those with one copy or controls [25]. Unfortunately we could not assess the effect of *APOE4* homozygoty in our sample, as only five participants carried a double copy of the ɛ4 allele. However, independently of *APOE4* status, lower REM-sleep percentage was significantly associated with higher wake after sleep onset, N1 sleep, and lower overall sleep efficiency in our sample. This suggests that although *APOE4* genotype only has a direct effect on REM sleep percentage, the reduction of REM sleep duration may be paralleled by other differences in sleep architecture, including greater amounts of wakefulness and lighter sleep.

The vulnerability of REM sleep to *APOE4* status is likely to result from ongoing early neurodegenerative processes. Indeed, REM sleep physiology and regulation critically depend on several nuclei located in the brainstem, hypothalamus, and basal forebrain [34]. However, subcortical nuclei implicated in REM-sleep physiology are affected early by tau-related neurodegeneration [35]. The locus coeruleus and basal forebrain are among the very first regions to accumulate tau pathology in the course of AD [36]. *APOE4* carriers present with a pattern of enhanced neurodegeneration in regions which are vulnerable to tau pathology, compared to non-carriers [37–39]. Cholinergic cells seem to be particularly affected and sensitive to tau pathology, and *APOE4* carriers show a greater rate of neurodegeneration in the nucleus basalis of Meynert over time compared to non-carriers [39], as well as more severe presynaptic cholinergic loss in targeted cortical areas [40, 41]. Given the implication of these nuclei in REM sleep physiology, and their sensitivity to tau-related neurodegeneration, it is not surprising to observe a vulnerability of REM sleep to *APOE4* genotype, which is the highest genetic risk factor for sporadic AD. Interestingly, we show here that the reduction of REM sleep in *APOE4* carriers was already detectable in cognitively unimpaired individuals. In fact, subcortical nuclei involved in REM-sleep physiology are affected by tau pathology at a very early stage, before medial temporal regions [36]. Future studies will need to investigate whether subcortical tau pathology drives REM-sleep alterations in *APOE4* carriers, even in older adults without cognitive deficits.

In addition, it is also plausible that REM-sleep alterations in *APOE4* carriers may partly result from white matter injury. Indeed, *APOE4* negatively affects lipid metabolism, which has been related to white matter injury [42]. Consistently, *APOE4* carriers exhibit alterations in white matter microstructure, as evidenced by diffusion imaging [43, 44]. Interestingly, lower REM sleep percentage has been associated with reduced white matter integrity in older adults, including lower fractional anisotropy and higher mean diffusivity in the corpus callosum [45]. REM sleep may therefore be more disrupted in *APOE4* carriers due to both tau-related neurodegeneration and white matter injury. Moreover, other pathways could partly explain why REM sleep is altered in *APOE4* carriers. *APOE4* has been shown to also affect synaptic plasticity, cerebrovascular health, neuroinflammation, and oxidative stress levels [46], which is relevant to REM sleep. Indeed, REM sleep is characterized by high metabolic levels and cerebral blood flow, and is important for synaptic remodeling and pruning, memory consolidation, and mood regulation [47]. Lower REM sleep duration has not only been associated with incident dementia [48] but also poorer mental health and increased overall mortality, including from cardiovascular causes [49, 50]. Interestingly, another study has shown that reduced REM sleep was associated with metabolic syndrome prevalence, but this association did not survive when accounting for multiple confounders and sleep-disordered breathing, suggesting that it was driven by comorbidities [51]. In our study, we excluded participants with neurological, psychiatric, and cerebrovascular diseases, which limited the impact of these potential confounders on REM sleep. However, it is still possible that the association between *APOE4* and REM sleep may be partly mediated by subclinical cardiovascular risk factors and synaptic dysfunction, which could ultimately increase the risk of cerebrovascular and mental health diseases, in addition to AD. Moreover, OSA did not significantly interact with *APOE4* in predicting REM sleep percentage. OSA is increasingly emerging as a potential risk factor for dementia [52] and is known to preferentially occur during REM sleep [53]. In our sample, OSA severity (as measured with the AHI) was significantly associated with lower REM sleep duration (*p* = .001), but the effects of OSA on REM sleep duration were not influenced by *APOE4* genotype. The link between OSA and *APOE4* is currently debated in the literature [54], but a meta-analysis performed on 6508 participants (including 1901 OSA cases) has concluded that *APOE4* carriers are not at increased risk of OSA compared to non-carriers [23]. Although longitudinal analyses should further confirm this result, this suggests that REM-sleep disruption in *APOE4* carriers is likely controlled by other mechanistic pathways than OSA, such as neurodegeneration in brainstem and basal forebrain nuclei, as hypothesized above.

Lastly, we found that age and sex did not significantly interact with *APOE4* status on the prediction of REM-sleep percentage. These observations are consistent with a recent study using data from the UK Biobank (*n* = 202, including 106 *APOE4* carriers), showing that *APOE4* homozygosity was related to greater informant-reported sleep disturbances when controlling for multiple demographic variables such as age, gender, depression, anxiety, and cognitive impairment [55]. Additional stratified analyses give preliminary insights on a potential higher vulnerability of REM sleep to *APOE4* genotype in older individuals and women (see Supplementary Material). However, trends were also present in younger individuals and men. These results warrant further replication in larger cohorts and should be interpreted with caution, given that no significant interactions were detected in the whole sample, that sample size was limited, and that inter-individual variability was high in some subgroups.

## Strengths and Limitations

Our study has the advantage of including a large sample of participants with PSG, and to investigate the impact of several potential modulating factors such as OSA, cognitive status, and demographic factors. However, some limitations must be mentioned. First, we were not able to test the impact of *APOE4* heterozygosity versus homozygosity. Indeed, we only had five participants with two copies of the ε4 allele in our sample. Second, we must acknowledge that as our sample included 41 *APOE4* carriers (20.7% of the whole sample), our sample size was relatively modest in some subgroups, albeit equally split (e.g. our sample included 21 women and 20 younger *APOE4* carriers), and inter-individual variability was also high in some subgroups. Therefore, statistical power may have impacted interaction and stratified analyses, and prevented us from testing the additive effect of confounding factors on sleep architecture. Third, we unfortunately could not assess the impact of AD biomarkers (i.e. amyloid and tau levels) on the association between *APOE4* genotype and REM sleep, and this should be investigated in future studies.

## Conclusions

In summary, we report that *APOE4* carriers exhibited a reduction in REM-sleep percentage, which was not influenced by age, sex, OSA, and cognitive status. This suggests that REM sleep is disturbed early in older participants at higher risk of dementia, even in the absence of cognitive deficits. Future studies will have to determine whether tau-related neurodegeneration in subcortical nuclei regulating REM sleep underlies its vulnerability to *APOE4* carriage.

## Supplementary Material

zsae094_suppl_Supplementary_Material

zsae094_suppl_Supplementary_Checklist

## Data Availability

Data used in the present study will be available from the corresponding author upon request.
