# Supplementary material for: Reduced rapid eye movement sleep in late middle-aged and older apolipoprotein E ɛ4 allele carriers
Source: Sleep. 2024 Apr 18;47(7):zsae094. doi: 10.1093/sleep/zsae094 (PMC11236949; doi:10.1093/sleep/zsae094)
Supplement: zsae094_suppl_Supplementary_Material [file zsae094_suppl_supplementary_material.docx]

**Supplementary Material**

**REM sleep is reduced in late middle-aged and older *APOE4* allele carriers**

Claire André^1,2^, Marie-Ève Martineau-Dussault^1,2^, Andrée-Ann Baril^1,3^, Nicola Andrea Marchi^1,2,4,5^, Véronique Daneault^1,2^, Dominique Lorrain^6,7^, Carol Hudon^8,9^, Célyne H. Bastien^8,9^, Dominique Petit^1,10^, Cynthia Thompson^1^, Judes Poirier^11,12^, Jacques Montplaisir^1,10^, Nadia Gosselin^1,2^, Julie Carrier^1,2^

1. Center for Advanced Research in Sleep Medicine, Hôpital du Sacré-Coeur de Montréal, Recherche CIUSSS NIM, Montreal, QC H4J 1C5, Canada.
2. Department of Psychology, Université de Montréal, Montreal, QC H2V 2S9, Canada.
3. Department of Medicine, Université de Montréal, Montreal, QC H3T 1J4, Canada.
4. Center for Investigation and Research in Sleep, Department of Medicine, Lausanne University Hospital and University of Lausanne, 1011 Lausanne, Vaud, Switzerland.
5. Laboratory for Research in Neuroimaging, Department of Clinical Neurosciences, Lausanne University Hospital and University of Lausanne, 1011 Lausanne, Vaud, Switzerland.
6. Research Centre on Aging, University Institute of Geriatrics of Sherbrooke, CIUSS de l’Estrie-CHUS, Sherbrooke, QC J1H 4C4, Canada.
7. Department of Psychology, Université de Sherbrooke, Sherbrooke, QC J1K 2R1, Canada.
8. CERVO Brain Research Centre, Québec City, QC G1E 1T2, Canada.
9. School of Psychology, Université Laval, Québec City, QC G1V 0A6, Canada.
10. Department of Psychiatry, Université de Montréal, Montréal, QC H3T 1J4, Canada.
11. Department of Psychiatry, McGill University, Montreal, QC H3A 1A1, Canada.
12. Douglas Mental Health University Institute, CIUSSS de l'Ouest-de-l'Ile-de-Montréal, Verdun, QC H4H 1R3, Canada.

**Corresponding author:**

Dr. Julie Carrier, PhD

Center for Advanced Research in Sleep Medicine, Hôpital du Sacré-Coeur de Montréal.

5400 Boul Gouin O, Montréal, QC H4J 1C5, Canada.

julie.carrier.1@umontreal.ca

**Table S1: Impact of *APOE4* status on REM sleep duration expressed in minutes.**

| **Variables** | **Sum of Squares** | **Degrees of freedom** | **F** | **p** | **η²_p_** |
| --- | --- | --- | --- | --- | --- |
| APOE4 status | 4866.59 | 1 | 9.23 | 0.003 | 0.047 |
| Age | 32.25 | 1 | 0.06 | 0.81 | 0.0003 |
| Sex | 37.97 | 1 | 0.07 | 0.79 | 0.0004 |
| Cognitive status | 311.31 | 1 | 0.59 | 0.44 | 0.003 |
| log(AHI) | 4698.64 | 1 | 8.91 | 0.003 | 0.045 |
| APOE4*Age | 6.53 | 1 | 0.01 | 0.91 | 0.0001 |
| APOE4*Sex | 73.52 | 1 | 0.14 | 0.71 | 0.001 |
| APOE4*Cognitive status | 1051.57 | 1 | 1.99 | 0.16 | 0.010 |
| APOE4*log(AHI) | 428.81 | 1 | 0.81 | 0.37 | 0.004 |
| *Residuals* | *99126.11* | *188* |  |  |  |

Type II ANCOVA showing the effect of APOE4 on REM sleep duration (expressed in minutes), and testing the interactions between *APOE4* status and covariates (i.e., age, sex, cognitive status and the AHI) on REM sleep duration in the whole sample.

*Abbreviations: AHI, apnea-hypopnea index; APOE4, ε4 allele of the Apolipoprotein E; η²_p_, partial eta squared; REM, rapid-eye movement.*

**Table S2: Impact of *APOE4* status on REM sleep proportion and duration, controlling for age, sex, cognitive status and REM-sleep apnea-hypopnea index.**

| **Variable** | **Sum of Squares** | **df** | **F** | **p** | **η²_p_** |
| --- | --- | --- | --- | --- | --- |
| **Dependent variable: REM-sleep percentage (% TST)** | | | | | |
| *APOE4* status | 290.55 | 1 | 8.93 | 0.003 | 0.045 |
| Age | 66.84 | 1 | 2.06 | 0.15 | 0.011 |
| Sex | 10.81 | 1 | 0.33 | 0.57 | 0.002 |
| Cognitive status | 2.86 | 1 | 0.09 | 0.77 | 0.0005 |
| log(REM-sleep AHI) | 326.48 | 1 | 10.04 | 0.002 | 0.051 |
| *APOE4**Age | 12.98 | 1 | 0.4 | 0.53 | 0.002 |
| *APOE4**Sex | 1.1 | 1 | 0.03 | 0.85 | 0.0002 |
| *APOE4**Cognitive status | 74.55 | 1 | 2.29 | 0.13 | 0.012 |
| *APOE4**log(REM-sleep AHI) | 75.56 | 1 | 2.32 | 0.13 | 0.012 |
| *Residuals* | *6114.25* | *188* |  |  |  |
| **Dependent variable: REM-sleep duration (min)** | | | | | |
| *APOE4* status | 4866.59 | 1 | 9.23 | 0.003 | 0.047 |
| Age | 32.25 | 1 | 0.06 | 0.81 | 0.0003 |
| Sex | 37.97 | 1 | 0.07 | 0.79 | 0.0004 |
| Cognitive status | 311.31 | 1 | 0.59 | 0.44 | 0.003 |
| log(REM-sleep AHI) | 4698.64 | 1 | 8.91 | 0.003 | 0.045 |
| *APOE4**Age | 6.53 | 1 | 0.01 | 0.91 | 0.0001 |
| *APOE4**Sex | 73.52 | 1 | 0.14 | 0.71 | 0.001 |
| *APOE4**Cognitive status | 1051.57 | 1 | 1.99 | 0.16 | 0.01 |
| *APOE4**log(REM-sleep AHI) | 428.81 | 1 | 0.81 | 0.37 | 0.004 |
| *Residuals* | *99126.11* | *188* |  |  |  |

Type II ANCOVA showing the effect of *APOE4* on REM sleep proportion (expressed as a percentage of total sleep time) or duration (expressed in minutes), and testing the interactions between *APOE4* status and covariates (i.e., age, sex, cognitive status and REM-sleep AHI) on REM sleep variables in the whole sample.

*Abbreviations: AHI, apnea-hypopnea index; APOE4, ε4 allele of the Apolipoprotein E; η²_p_, partial eta squared; REM, rapid-eye movement; TST, total sleep time.*

**Table S3: Associations between REM sleep percentage and other sleep architecture variables.**

| **Predictor** | **Unstandardized coefficient (95% CI)** | **Standard Error** | **Standardized**  **coefficient** | **p** |
| --- | --- | --- | --- | --- |
| **Whole sample (n=198)** | | | | |
| Total sleep time (min) | 0.03  (0.02 – 0.04) | 0.006 | 0.33 | **<0.001** |
| Sleep efficiency (%) | 0.21  (0.15 – 0.27) | 0.03 | 0.44 | **<0.001** |
| Log(Nb of awakenings) (nb) | -4.29  (-8.51 – -0.07) | 2.14 | -0.17 | 0.046 |
| Log(WASO) (min) | -8.22  (-11.35 – -5.10) | 1.59 | -0.35 | **<0.001** |
| Log(N1 sleep) (% TST) | -5.965  (-10.10 – -1.83) | 2.10 | -0.25 | **0.005** |
| N2 sleep (% TST) | -0.08  (-0.17 – 7.76×10-4) | 0.04 | -0.14 | 0.052 |
| Log(N3 sleep) (% TST) | -2.10  (-4.06 – -0.13) | 0.996 | -0.17 | 0.036 |
| **APOE4 carriers (n=41)** | | | | |
| Total sleep time (min) | 0.04  (0.02 – 0.10) | 0.009 | 0.52 | **<0.001** |
| Sleep efficiency (%) | 0.19  (0.09 – 0.29) | 0.05 | 0.55 | **<0.001** |
| Log(Nb of awakenings) (nb) | 1.62  (-6.65 – 9.90) | 4.07 | 0.08 | 0.69 |
| Log(WASO) (min) | -5.89  (-12.34 – 0.56) | 3.18 | -0.31 | 0.07 |
| Log(N1 sleep) (% TST) | -2.82  (-11.95 – 6.31) | 4.50 | -0.14 | 0.55 |
| N2 sleep (% TST) | -0.02  (-0.20 – 0.15) | 0.08 | -0.05 | 0.77 |
| Log(N3 sleep) (% TST) | -3.01  (-7.32 - 1.31) | 2.12 | -0.29 | 0.17 |

Multiple regression analyses between sleep architecture variables as predictors, and REM sleep percentage as the dependent variable, controlling for age, sex, cognitive status and the AHI in the whole sample (n=198), and in *APOE4* carriers only (n=41). Results in bold survived a Bonferroni correction for multiple comparisons (p=0.05/number of comparisons=0.05/7=0.007).

*Abbreviations: AHI, apnea-hypopnea index; APOE4, ε4 allele of the Apolipoprotein E; CI, confidence interval; Nb, number; REM, rapid-eye movement; TST, total sleep time; WASO, wake after sleep onset.*

**Figure S1: Differences in REM sleep proportion according to *APOE4* status in sub-groups stratified for age, sex, cognitive status and OSA diagnosis.**

**
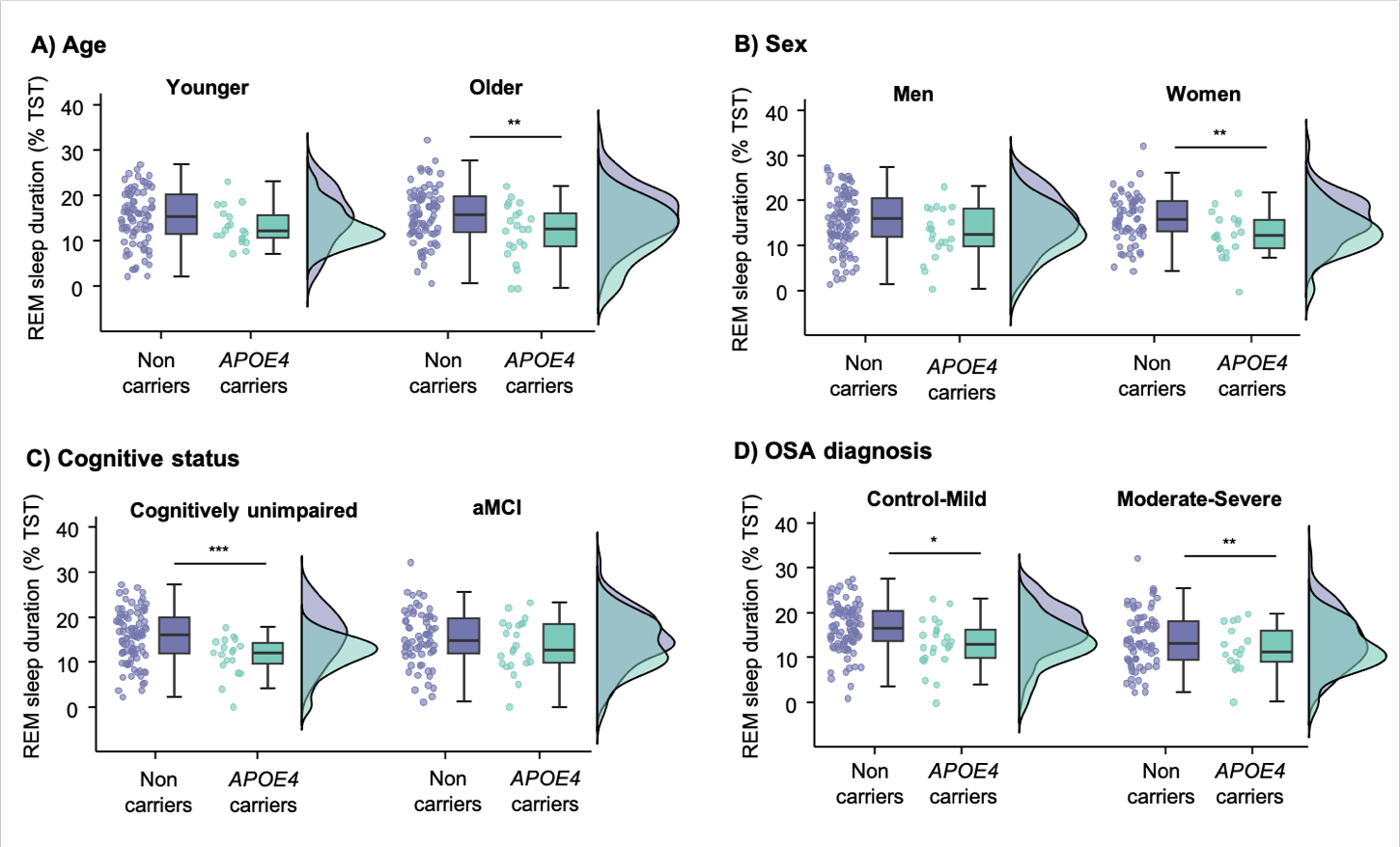
**

Raincloud plots showing REM sleep proportion values according to *APOE4* status in sub-groups stratified by A) age, B) sex, C) cognitive status and D) OSA diagnosis. Boxplots represent medians, interquartile range and associated confidence intervals. ANCOVAs tested the effect of *APOE4* status on REM sleep percentage in each sub-group, controlling for the remaining appropriate covariates. * p<0.05, ** p≤0.01, *** p≤0.001.

*Abbreviations: APOE4, ε4 allele of the Apolipoprotein E; OSA, obstructive sleep apnea; REM, rapid eye movement; TST, total sleep time.*

**Table S4: Impact of *APOE4* status on REM sleep proportion in sub-groups stratified for age, sex, cognitive status and OSA diagnosis**

| **Group** | **Predictor** | **Sum of Squares** | **Degrees**  **of freedom** | **F** | **p** | **η²_p_** |
| --- | --- | --- | --- | --- | --- | --- |
| **Age-stratified sub-groups** | | | | | | |
| Younger | *APOE4* status | 98.128 | 1 | 3.454 | 0.066 | 0.036 |
| Older | *APOE4* status | 298.525 | 1 | 8.471 | **0.005** | 0.083 |
| **Sex-stratified sub-groups** | | | | | | |
| Men | *APOE4* status | 139.246 | 1 | 3.734 | 0.056 | 0.032 |
| Women | *APOE4* status | 187.532 | 1 | 7.069 | **0.010** | 0.085 |
| **Sub-groups stratified by cognitive status** | | | | | | |
| Cognitively unimpaired | *APOE4* status | 341.838 | 1 | 11.736 | **<0.001** | 0.101 |
| aMCI | *APOE4* status | 62.583 | 1 | 1.731 | 0.192 | 0.020 |
| **Sub-groups stratified by OSA diagnosis** | | | | | | |
| Control-Mild | *APOE4* status | 241.623 | 1 | 8.141 | **0.005** | 0.071 |
| Moderate-Severe | *APOE4* status | 138.127 | 1 | 4.094 | **0.046** | 0.049 |

Type II ANCOVAs showing the effect of *APOE4* on REM sleep proportion (expressed as a percentage of total sleep time) in sub-groups stratified by age, sex, cognitive status and OSA diagnosis, controlling for age, sex, cognitive status and OSA diagnosis as appropriate (e.g., sex-stratified analyses were controlled for age, cognitive status and the AHI). For age, we separated the sample into a “younger” (n=98 participants including 19 *APOE4* carriers; age range: 55-68 years old, mean age: 61.9 ± 4.2 years) and “older” (n=100 participants including 22 *APOE4* carriers; age range: 69-86 years old, mean age: 75.35 ± 4.8 years) group based on a median split on age. For OSA levels, we separated the sample based on an AHI≥15.

*Abbreviations: AHI, apnea-hypopnea index; APOE4, ε4 allele of the Apolipoprotein E; MCI, Mild Cognitive Impairment; η²_p_, partial eta squared; OSA, obstructive sleep apnea; REM, rapid-eye movement; TST, total sleep time.*
